# Isolated pancreatic heamorrhage in association with anticoagulation

**DOI:** 10.1186/1477-9560-11-20

**Published:** 2013-10-01

**Authors:** Keith Chiu, Abdul Razack, Anthony Maraveyas

**Affiliations:** 1 Radiology Department, Hull and East Yorkshire NHS Trust, Hull Royal Infirmary, Anlaby Road, Hull HU3 2JZ, UK; 2 The Queen’s Centre for Oncology and Haematology, Hull and York Medical School, Castle Hill Hospital, Castle Road, Hull HU16 5JQ, UK

## Abstract

Haemorrhage is the primary complication of anticoagulation therapy with the gastrointestinal, urinary and nasal tracts the most common sites of bleeding. Haematoma within solid organs is uncommon especially in the absence of blunt trauma. We describe two patients on long term Warfarin therapy who developed focal haematomas within the pancreas. To the best of our knowledge these are the first isolated unprovoked focal pancreatic hematoma cases reported in the literature. The non-specific clinical symptoms and confusing radiological features mimicked pancreatic malignancy and this led to misdiagnosis in the one patient who underwent unnecessary surgical exploration. The haematoma was correctly identified in the second patient who was managed conservatively and had an uneventful recovery.

## Background

Anticoagulation therapy is essential in preventing many thromboembolic events and warfarin is the most commonly prescribed oral anticoagulation therapy. Unfortunately, the pharmacokinetics and pharmacodynamics of Warfarin are complex and its usage requires regular monitoring. Haemorrhagic complications associated with warfarin can cause significant morbidity and mortality to patients [1].

We report two cases of isolated pancreatic haemorrhage mimicking pancreatic malignancy and discuss the challenges in correctly diagnosing these two cases as well as how to differentiate pancreatic malignancy and haemorrhage from its mimics.

## Case presentation

The first patient was a 66 year-old male who presented acutely to the surgical admissions unit in January 2011. The patient had 4 episodes of fresh rectal bleeding within 48 hours and non-specific abdominal pain. The patient had had mechanical aortic valve replacement four years previously for aortic stenosis. The patient had also suffered from abdominal aortic aneurysm rupture and had undergone an emergency open abdominal aorta repair three years prior to this admission. The patient was on multiple medications including amiodarone, perindopril and long term Warfarin therapy.

**Figure 1:**
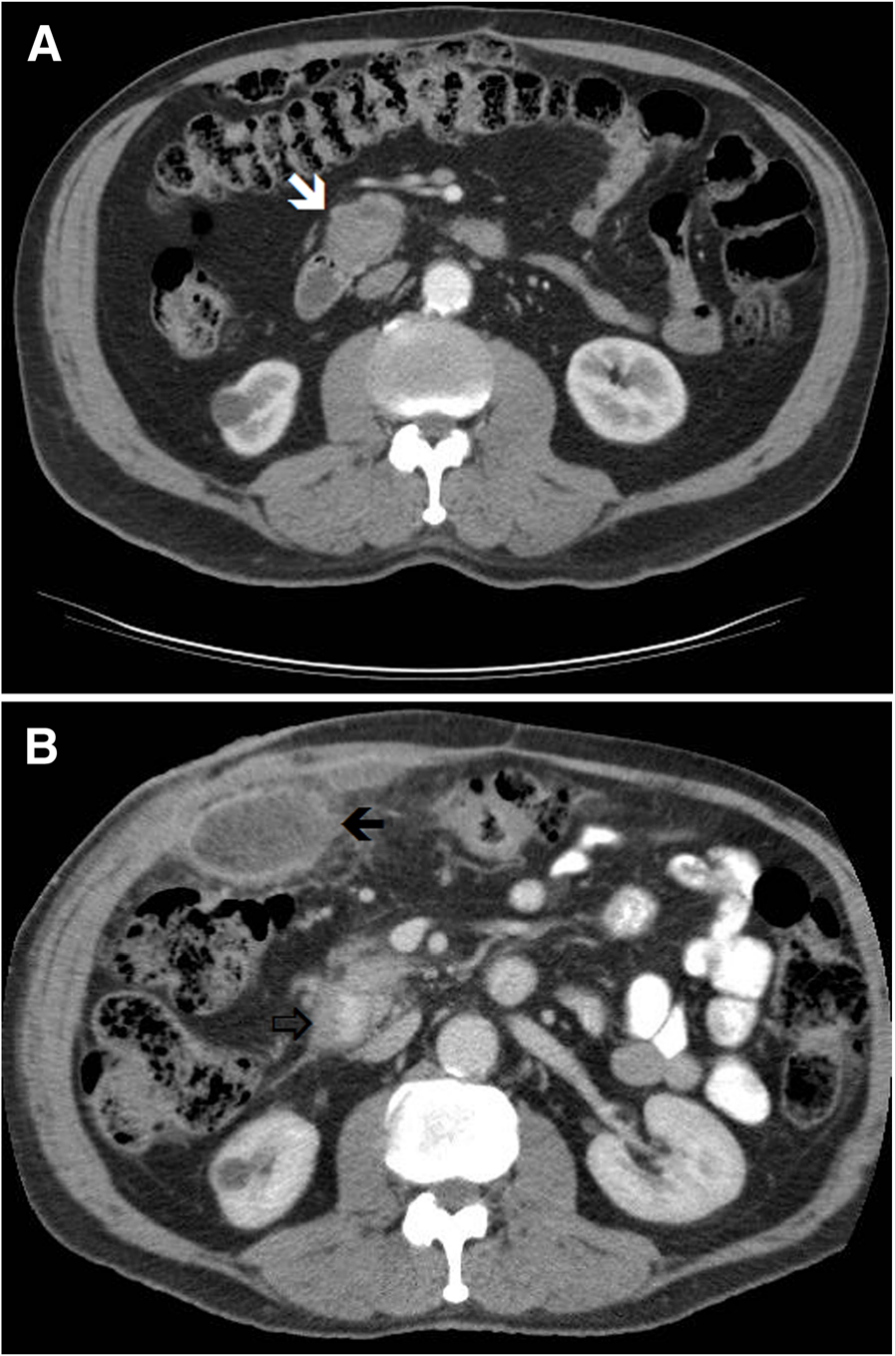
**CT images of the first patient. (A)** An 3 cm lesion was seen on initial post contrast venous phase CT (white arrow). This was mis-interpreted as a pancreatic malignancy. **(B)** Patient underwent a laparotomy which failed to identify a mass in the pancreas. Post operatively, he developed a wound site abscess (black arrow).

On admission, the patient had a raised serum lactate of 5.0 mmol/L, white cell count of 17.6 × 10^12/L (neutophilia), a chronic normocytic anaemia of 9.4 g/dl but no acute drop in haemoglobin level. The patient’s anticoagulation was well controlled and had an international normalised Ratio (INR) of 3.1 that was within therapeutic range for the patient. Other blood examination was normal. The surgical team was concerned that there may be an aorto-enteric fistula in view of previous aortic surgery. A Computed Tomography (CT) scan was carried out and an unexpected 3 cm mass in the head of pancreas was identified (Figure 1A). The patient underwent an oesophagogastroduodenoscopy (OGD) and a colonoscopy which were normal. The case was referred to the hepato-pancreatico-biliary multidisciplinary meeting (MDT) and the lesion was presumed to be a potentially operable pancreatic adenocarcinoma. An MRI had been suggested for further assessment of this lesion but this was overlooked and decision was made for trial dissection with a view to perform a curative Whipple’s procedure. In July 2011, the patient underwent a laparotomy and intra-operatively, the hepatobiliary surgeon was unable to identify the pancreatic lesion. An intra-operative ultrasound was carried out by two experienced cross-sectional radiologists that also failed to identify the lesion. The laparotomy was abandoned.

The patient was clinically septic during the post-operative period and a follow-up CT scan 2 weeks post-surgery showed that the pancreatic lesion had resolved completely (Figure 1B). The patient went on to develop an abscess in the wound site. He had debridement and wash-out of the abscess under general anesthesia and made an uneventful recovery thereafter.

The second patient was a 67 year-old female who was admitted to the medical admissions unit in November 2011 with abdominal pain. The patient had mitral valve repair nine months prior for mitral stenosis and had been on Warfarin therapy since. The patient also suffered from rheumatoid arthritis and was on methotrexate and prednisolone. Initial blood examination showed a raised white cell count of 17.6 × 10^12/L and a normocytic anaemia of 10.9 g/dl. Patient’s anticoagulation was poorly controlled since beginning Warfarin therapy and on admission had an INR of 9.5.

**Figure 2:**
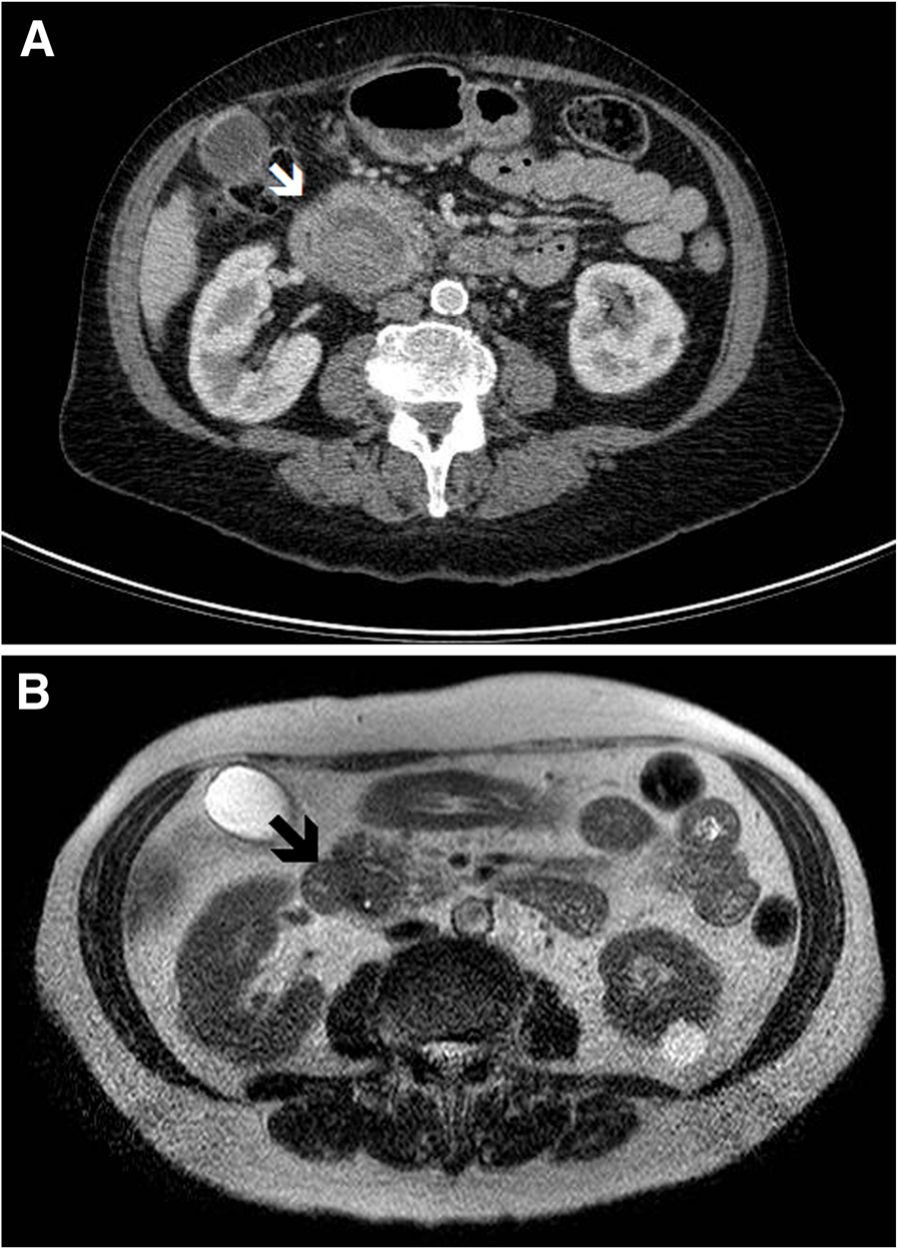
**CT and MRI images of the second patient. (A)** A large heterogenous mass (white arrow) was identified at the head of pancreas. This was identified as a haematoma. **(B)** Follow-up MRI showed the pancreatic mass (black arrow) had decreased in size and demonstrated characteristic signals for a haematoma.

The patient’s anticoagulation therapy was stopped and INR reversed back to normal while put on intravenous heparin. She also underwent an ultrasound scan that demonstrated a large heterogeneous mass arising from the pancreatic head. A CT scan was then carried out that confirmed the presence of a pancreatic mass which was thought to be a malignant lesion (Figure 2A).

The case was discussed in the hepato-pancreatico-biliary MDT and on review the lesion was thought to be a haematoma rather than a malignant lesion. A decision to ‘watch and wait’ was adopted. The patient’s symptoms settled with conservative management and was discharged home. An interval Magnetic Resonance Imaging (MRI) scan one month after discharge showed the pancreas mass had decreased in size and showed MR characteristics of a haematoma (Figure 2B). A further follow-up CT 3 months later showed complete resolution of the pancreatic mass. The patient was discharged from the care of the hepato-pancreatico-biliary service.

## Discussion

To our knowledge, this is the first descriptions of isolated focal haemorrhage in the pancreas as a result of oral anticoagulation. The periampullary region is anatomically and physiologically complex, thus focal haemorrhage involving any of its structures can mimic the presentation of a neoplasm. Although at times challenging, they can be differentiated by various imaging modalities from their enhancement pattern, signal characteristics or metabolic activities [2,3]. What is unique in these two cases is that the patients had isolated focal pancreatic parenchymal haemorrhage in the absence of a neoplasm, pancreatitis or trauma.

Perhaps it is not surprising that our two cases were initially misinterpreted as pancreatic malignancies as the presence of a pancreatic mass is the most common direct sign of a pancreatic tumour. The most common type of pancreatic cancer, accounting for over 85% of these tumours is ductal adenocarcinoma. They often cause distortion to the contour of the gland, dilated pancreatic and common bile ducts, atrophy of the gland, invasion of local vasculature as well as metastases to regional lymph nodes, liver and the peritoneal cavity [4]. However, haemorrhage can also occur within a pancreatic tumour and is common in neoplasms such as solid pseudopapillary tumours and acinar cell carcinoma [5,6]. Therefore, only by recognizing the lack of indirect features, other lesions such as haematomas can be distinguished from a pancreatic tumour.

Tumoural haemorrhage is not only cause of haemorrhage within the pancreas. Pancreatic haemorrhage is also often associated within pancreatitis, occuring in 20% of cases [7]. Although serum amylase is the most widely used biochemical test to diagnose acute pancreatitis [8], CT plays an ever increasing role in aiding diagnosis and predicting clinical outcome [9]. In general, haemorrhage associated with pancreatitis also exhibit other signs of pancreatitis, including focal or diffuse pancreatic enlargement, pseudoaneurysms, inflammatory changes in the surrounding peripancreatic space, fluid collection formation and necrosis of the parenhcyma. The absence of these signs was not recognized which resulted in the misinterpretation of the radiological findings. If the CT scan had been performed with a non-contrast phase followed by arterial and venous phase study, the high Hounsfield (HU) values on the non-contrast images along with the lack of increase in HU values following contrast injection would have helped in making the correct diagnosis.

Other causes of pancreatic haemorrhage include pancreatic injury secondary to trauma or iatrogenic cause (such as biopsy) [10,11]. In these circumstances, although radiological appearances may be similar to these two cases, there is usually a clear history for making the diagnosis.

The two cases illustrate the potential morbidity of anticoagulation therapy. It is estimated that around 1% of the UK population is on oral anticoagulation therapy (source IMS Health) and haemorrhage is the most common complication of Warfarin therapy [12]. The risk of major bleeding is thought to be around 2% in clinical trials although the incidence of major haemorrhage is unknown due to the varying dosage used, patient characteristics and differential definitions used by various studies [13]. Multiple factors such as the intensity of anticoagulation, time from starting anticoagulation therapy, quality of monitoring, genetic factors, age, gender, co-morbidities, polypharmacy and patient compliance have all been identified to be associated with anticoagulation bleeding complications [14].

The importance of recognizing the potential complications of anticoagulation therapy cannot be understated. Only by increasing the awareness amongst physicians, surgeons and radiologists, unnecessary procedures as in one of our patients who underwent laparotomy and had a prolonged hospital stay could be avoided.

## Conclusion

Haemorrhage secondary to anticoagulation therapy is common although focal haemorrhage in a solid organ is rare. These two cases provide a timely reminder of the potential complications of anticoagulation therapy. Careful analyses of radiological images and an awareness of the complications of anticoagulation therapy can reduce morbidity of patients.

## Consent

Written informed consent was obtained from the patients for publication of this case report and any accompanying images. A copy of the written consent is available for review by the Editor-in-Chief of this journal.

## Competing interests

The authors declare that they have no competing interests.

## Authors’ contributions

AM and AR were involved in the management of the patient. KC prepared the initial manuscript. All three authors reviewed and approved the final manuscript.
